# Simulating denial increases false memory rates for abuse unrelated information

**DOI:** 10.1002/bsl.2566

**Published:** 2022-02-22

**Authors:** Charlotte A. Bücken, Ivan Mangiulli, Henry Otgaar

**Affiliations:** ^1^ Faculty of Law Leuven Institute of Criminology KU Leuven Belgium; ^2^ Forensic Psychology Section Faculty of Psychology and Neuroscience Maastricht University Maastricht The Netherlands

**Keywords:** false denial, false memory, misinformation, simulated memory error, victim memory

## Abstract

Victims of abuse might deny their traumatic experiences. We studied mnemonic effects of simulating false denial of a child sexual abuse narrative. Participants (*N = *127) read and empathized with the main character of this narrative. Next, half were instructed to falsely deny abuse‐related information while others responded honestly in an interview. One week later, participants received misinformation for the narrative and interview. In a final source memory task, participants' memory for the narrative and interview was tested. Participants who falsely denied abuse‐related information endorsed more abuse‐unrelated misinformation about the event than honest participants. Abuse‐related false memory rates did not statistically differ between the groups, and false denials were not related to omission errors about (1) the interview and (2) narrative. Hence, victim's memory for abuse‐related information related to their experience might not be affected by a false denial, and inconsistencies surrounding the *abuse‐unrelated* information are more likely to take place.

## 1 INTRODUCTION

Victims of (child) sexual abuse might deny their traumatic experiences. While there is currently a discussion surrounding the prevalence of false denials in child sexual abuse investigations (London et al., 2020; Lyon et al., 2020), there is agreement that children often delay disclosure, and might initially deny that any abuse took place before coming forward (Eisen et al., 2021; London et al., 2020). When they eventually talk about the abuse, these victim's memory statements serve as important pieces of evidence in court and can influence decisions surrounding culpability (Romeo et al., 2018). This is especially relevant in child sexual abuse cases because there is often a lack of physical evidence (e.g., injuries) related to the offense so that testimonies of victims and perpetrators are frequently the only pieces of evidence available (Johnson et al., 2017).

It is therefore vital that victim's reports are as accurate and complete as possible. However, when children are reluctant to disclose, and the police has a pre‐conceived idea of what ostensibly happened, the interviewer might resort to suggestive tactics that can ultimately result in false reporting undermining the validity of children's testimonies (Lamb et al., 2002). Statement inconsistencies, for example, because the victim forgets information about the event or what they discussed in previous interviews, further reflect negatively on credibility in the eyes of legal stakeholders (Fisher et al., 2013). Moreover, lying has been associated with memory distortions (for a review, see Otgaar & Baker, 2018). Thus, it is vital to understand how false denials might affect the victim's memory record for (1) the event and (2) the repetitive interviewing process that such victims often go through (e.g., Lamb et al., 2002; Otgaar & Baker, 2018). However, the area of denials and memory is still understudied.

### 1.1 Mnemonic effects of false denials

Several studies have examined the mnemonic impact of false denials from a witnessing perspective (Battista et al., 2021, 2020; Otgaar et al., 2016, 2020, 2014; Romeo et al., 2019). These studies usually have followed the following paradigm. First, participants viewed word lists, pictures, or videos. Second, all participants engaged in a memory test, in which the experimenter interviews them about their memory for the initially viewed stimuli. Here, some participants are asked to falsely deny information (e.g., “I did not see…”), while others are asked to be honest. After a delay, participants' memory for both the initial stimulus and interview is tested in a source memory test. The canonical finding is that participants who falsely denied information forget which information they lied about in the interview (i.e., second phase of the experiment; e.g., Otgaar et al., 2014, 2016, 2018; Romeo et al., 2019). This finding has been termed “denial‐induced forgetting” (i.e., DIF; Otgaar et al., 2016).

Theoretically, retrieval inhibition, or memory suppression, has been proposed as the mechanism driving the memory undermining effects of false denial (Otgaar & Baker, 2018; Otgaar et al., 2020). Imagining that memory works like a network of associatively related concepts (i.e., “details,” or “nodes”; Howe et al., 2009), Otgaar et al. (2020) argued that after a person has falsely denied certain details, they become less accessible at retrieval for a certain period of time for the rememberer, perhaps due to inhibition of the denied information.

Moreover, the authors argued that this retrieval inhibition could even spread to related nodes in the memory network that were not actually part of the experienced event. To examine this, Otgaar et al. (2020) conducted two experiments on the impact of false denials on the generation of false memories—memories for details or events that never actually happened (Otgaar et al., 2020). In these experiments, Otgaar et al. (2020) examined false memories that arise endogenously (i.e., spontaneously) by using Deese‐Roediger‐McDermott (DRM) word lists (Deese, 1959; Roediger & McDermott, 1995). DRM word lists contain associatively related words (e.g., “jazz,” “guitar,” “dance,” “drum,” and “listen”) that are linked to a related, but unpresented critical lure word (i.e., “music”). A recurrent finding is that a non‐trivial percentage of participants falsely report the critical lure (Roediger & McDermott, 1995). Interestingly, Otgaar et al. (2020) found that participants who were instructed to falsely deny words from the DRM lists in the interview produced fewer false memories for the initially presented word lists compared to controls. Therefore, the authors concluded that perhaps retrieval inhibition spread to these related but unpresented critical lures.

### 1.2 Effects of denial on event memory

Although DIF usually pertains to the time at which the denial takes place, some studies additionally found an effect of false denials on memory for the stimulus itself (Battista et al., 2021; Otgaar et al., 2020; Romeo et al., 2019; Vieira & Lane, 2013). Because this effect on the stimulus has not always been replicated, it is unclear whether these were false positive results, or something else was at play. For example, Battista et al. (2021) examined whether cognitive resources affected the impact of denials on memory. Their study used a DIF paradigm in which participants were instructed to (1) deny all details of the event (simple denial), (2) deny only part of the events while being honest about others (complex denial), or to (3) respond honestly. They replicated DIF for the interview and additionally found that complex denials also lead to a forgetting effect that applies to the stimulus. Relatedly, research has also found a forgetting effect for the experienced stimulus when using negative emotional or traumatic events (Romeo et al., 2019). Here, the authors argued that participants might have tried not to think about the aversive events, thus leading to an increased inhibition effect (Romeo et al., 2019).

### 1.3 Denials and post‐event information

It is imperative to realize that when a potential victim is being interviewed, the police already has acquired other information on the case (i.e., from other witnesses) and has created a (mental) script of what they think unfolded (Goodman‐Brown et al., 2003). Especially when faced with a reluctant victim, the interviewer might resort to asking whether specific details took place that are in line with this script to facilitate disclosure (Lamb et al., 2002). Yet, these questions might accidentally contain misleading post‐event information (i.e., misinformation).

Apart from interviewing settings, victims of abuse could also be exposed to misinformation in other contexts. That is, in some abuse cases there is more than one victim, or other witnesses are present. Such co‐witnesses oftentimes talk with each other (Paterson & Kemp, 2006), but a reluctant victim might also receive information from other victims or witnesses through secondary sources (e.g., family members). The police might also provide a summary of previous interviews, and this can accidentally include false information (Cochran et al., 2016; Sagana et al., 2014). Thus, a potential victim might come into contact with misleading information about both the event and the interviewing process.

This misinformation can eventually lead to false reporting and suggestion‐based false memories (Loftus, 2005). These false memories differ from spontaneous ones because they arise after external influence (e.g., suggestion or misinformation). In the typical misinformation paradigm, a three‐step procedure is employed. First, participants are presented with a stimulus (e.g., a video or pictures). Second, they receive misinformation (e.g., “Did you see a yield sign?”, when the picture actually depicted a stop sign). Third, participants' memory for the stimulus is tested. The canonical finding is that presenting misinformation can lead participants to report this false information; an effect called the *misinformation effect* (Loftus, 2005). Since there are several routes through which a (falsely) denying victim might come into contact with misinformation, it is relevant to investigate the effect of false denials on misinformation endorsement.

### 1.4 Theoretical considerations

What would we expect from a theoretical perspective when someone who has (falsely) denied that an event happened is faced with misinformation? A popular theory to explain false memories is Fuzzy Trace Theory (Reyna & Brainerd, 1998). Fuzzy Trace Theory postulates that any experienced event is stored in two independent memory traces: a gist trace and a verbatim trace (Brainerd et al., 2008; Reyna & Brainerd, 1998). Gist traces store general information related to the underlying meaning of an experience (e.g., the theme of an event). Verbatim traces store item‐specific, detailed information of an experience. According to the authors of this theory, verbatim traces fade (i.e., are forgotten) more quickly than gist traces. If verbatim traces are then not available at retrieval, people rely on gist traces to “fill in the blanks” in memory. Because gist traces only store the underlying meaning of an event, false information is endorsed more when someone relies on gist than verbatim traces. If false denials really are related to forgetting, then—drawing on the tenets of Fuzzy Trace Theory—we might expect that false denials are related to a more rapid deterioration of verbatim memory traces. Therefore, after a false denial a person would have to rely more heavily on gist traces and thus be more prone to endorsing misinformation compared with a consistently honest person whose verbatim traces might be more intact.

Another memory principle that would support the same prediction is the Discrepancy Detection Principle (Tousignant et al., 1986). This principle holds that the endorsement of misinformation depends on whether someone notices that there is a difference—a discrepancy—between the experienced event and the received post‐event information. Specifically, someone who has not noticed that the post‐event information does not match with what truly happened (i.e., because of forgetting) is more likely to endorse misinformation than someone who *has* noticed this mismatch. Hence, if false denials are related to forgetting, someone who has falsely denied information would be less likely to detect this discrepancy, and thus be more likely to endorse misinformation compared with a consistently honest person. Mangiulli et al. (2020) followed this same line of argumentation in their paper on feigned amnesia (i.e., say that they do not remember details) of a crime—which is another lying strategy known to undermine memory—and the misinformation effect. However, in their experiment feigning amnesia did not influence the misinformation effect and Mangiulli et al. (2020) argued that certain limitations related to their design might have been the reason (e.g., the timing of the misinformation and type of memory test used).

However, Associative‐activation Theory (Howe et al., 2009; Otgaar et al., 2019) would lead to a different interpretation. Associative‐activation theory postulates that representations of our memories are encoded in a system of related knowledge nodes (i.e., concepts such as “chair” or “wooden”) that have associations with each other. The strength of associations depends on how often nodes are activated together. When a memory is encoded, the activation of concepts relevant to this memory spreads through the memory network. This associative activation can be quite fast and automatically lead to the activation of strongly related nodes that were not part of the experienced event. Such activation can in turn lead to false memories (Howe et al., 2009). Misinformation that is associatively related to the experienced event might thus be activated when the event is encoded and lead to false memories. However, this might not happen if false denials induce retrieval inhibition. This inhibition may also prevent activation from spreading to other related nodes (e.g., associatively related misinformation) and thus limit false memory production, as argued by Otgaar et al. (2020) who looked at false denials and spontaneous false memories. Indeed, then we might expect that false denials could lead to lower misinformation endorsement (i.e., false memories) after false denial.

### 1.5 Witnessing versus victim perspective

An important research gap in this previous work on false denial relates to the fact that most work has been conducted from a witness perspective (for a summary, see Otgaar & Baker, 2018). Yet, denials also occur quite frequently in victim cases. Indeed, a recent study found that among 132 child victims involved in forensic evaluations, 39% (*n* = 29/74) of children in substantiated sexual abuse cases, and 55.6% (*n = *35/78) of children in substantiated physical abuse cases denied their abuse in at least one interview with authorities (Eisen et al., 2021). Of importance, there is reason to believe that memory is differentially affected from a bystander's than a victim's perspective. Specifically, self‐performed actions are better remembered compared with actions we watch someone else perform, also known as the *action superiority effect* (Engelkamp & Cohen, 1991; Engelkamp et al., 2004). A recent study found that even after false denials, participants do not forget that they performed certain actions (Bücken et al., 2022) showing the memory strength of self‐performed actions. Furthermore, when directly experiencing an event as a victim, something called the self‐reference effect likely sets in which stipulates that information related to the self is remembered better than information related to someone else (Symons & Johnson, 1997). Yet, the mnemonic effects of false denials have never been studied from a victim perspective.

McWilliams et al. (2014) devised a paradigm to examine the victim perspective in an ethically sound way also labeled the simulated memory error paradigm. In their experiment, participants were instructed to read twice a case vignette describing an incident of child sexual abuse in the second person, and to mentally role‐play taking the part of the victim. After a delay, participants recalled the events from the first‐person perspective honestly, while simulating a memory error, or did not rehearse the story. One week later, all participants recalled the narrative truthfully.

McWilliams et al. (2014) found that compared to truthful recall, untruthful recall or no rehearsal in session one impaired memory for the narrative. The simulated memory error paradigm has since been successfully employed in other experiments to study memory from the victim perspective (e.g., Hong & Hobbs, 2021; Newton & Hobbs, 2015). While other memory errors (e.g., omission, confabulation, and blame‐attributions) have been studied in this paradigm (Hong & Hobbs, 2021; McWilliams et al., 2014; Newton & Hobbs, 2015), it has never been used to study the mnemonic effects of false denials. Hence, the SMEP is a promising method to study the effect of false denials on victims' memory in a controlled laboratory setting.

### 1.6 The current experiment

The current study aimed to examine the impact of false denials on forgetting and misinformation endorsement in the simulated memory error paradigm. Thereby, we attempted to replicate the findings of false denial research from a victim perspective, and extended them by including misinformation. Specifically, in our experiment, participants were asked to read and roleplay a child sexual abuse scenario. Subsequently, those in the false denial group were instructed to honestly remember non‐abuse portions of the narrative, but to deny abuse‐related information in (1) a free recall task and (2) a written “interview,” in which specific yes/no type questions were asked. Participants in the honest control condition were instructed to respond honestly. After a 1‐week delay, participants received misinformation about the narrative and the previous memory interview. Finally, participants engaged in a source memory test.

Our prediction was that we would find DIF for the phase during which the denial occurred. Yet, the current experiment employed the victim perspective, which might render it less likely to find DIF (as argued above) so that it was ultimately an empirical question whether the effect could be replicated. Furthermore, we expected to find that falsely denying participants would result in more omission errors regarding the narrative compared with honest control participants. This is because in the current experiment we aimed to mirror the situation in real cases by (1) employing a complex denial—participants falsely denied abuse related information while correctly recalling abuse unrelated information—of an (2) emotionally negative event (i.e., child sexual abuse). Previous research has shown that both of these elements (i.e., complexity of the denial and emotionality of the event) can contribute to the effects of false denials on memory for the stimulus (Battista et al., 2021; Romeo et al., 2019).

Lastly, we expected the following regarding misinformation endorsement. If false denials lead to weakened memory—of the interview or narrative (Otgaar & Baker, 2018; Otgaar et al., 2020), there are two possible scenarios. Based on Fuzzy Trace Theory and the Discrepancy Detection Principle (Reyna & Brainerd, 1998; Tousignant et al., 1986), we would expect that participants in the false denial group endorse *more* misinformation than honest participants. That is, Fuzzy Trace Theory would suggest that false denials—due to their forgetting effects—could be associated with increased reliance on and retrieval of gist traces, thereby increasing false memories. The Discrepancy Detection Principle might postulate that false denials reduce discrepancy detection because information is forgotten, and thus render it more likely that misinformation is endorsed. Alternatively, and in line with the Associative‐activation Theory (Howe et al., 2009), we might expect that participants in the false denial group endorse *less* misinformation than honest participants, if retrieval inhibition caused by the denial also indirectly affects the activation of associatively related misinformation, by decreasing the activation of this related information.

## 2 METHOD

### 2.1 Participants

Participants were criminology bachelor students taking the course “Criminological Psychology” and psychology bachelor students at KU Leuven. Participants joined the experiment as part of their course assignment, on a voluntary basis or for partial course credit, and were recruited in class and online (i.e., through the university‐based online experimental sign up system of KU Leuven, SONA). An a priori power analysis using G*Power (Faul et al., 2007; i.e., test family: *t*‐test, difference between two means, *d* = 0.5, *α* = 0.05, power = 0.80) based on previous work (Houben, 2016; Otgaar et al., 2016, 2018, 2020; Romeo et al., 2019) indicated that a sample of 128 participants would be needed.

We collected data from 189 participants. Thirty‐seven participants did not complete both sessions and were therefore excluded from the analyses. Of the remaining 152 participants, 25 did not follow the instructions properly (e.g., they responded honestly or completely omitted the abuse even though they were asked to deny in response to specific questions). These were therefore excluded from our analyses. Lastly, we embedded six attention checks in the experiment. Participants who failed one of these but followed the instructions properly were included in the final sample. Two participants failed two of six attention checks, all pertaining to one specific questionnaire (i.e., the COPE; see below). Checking the rest of their data, these participants followed the instructions properly and answered in detail, so that their answers were included for the main memory related analyses. Thus, our final sample for the main analyses was *N* = 127 (randomly assigned to the denial condition: *n* = 50, honest control condition: *n* = 77). Participants were on average 19.23 years old (Range = 18–47, *SD* = 2.8) and were predominantly female (89%, *n* = 113). Ethical approval was obtained at KU Leuven from the Social and Societal Ethics Committee (reference: G‐2021‐3093‐R2(MAR)). The experiment was preregistered on the Open Science Framework (OSF; https://osf.io/gtuwj/?view_only=b508d059d241452d9546c92fc5b8739c) and all materials, data, and additional exploratory analyses are also accessible there (https://osf.io/9c5j4/?view_only=dcb3d7c42fb24e33afc030f326294061).

### 2.2 Materials

#### 2.2.1 Session 1: Child sexual abuse narrative

The child sexual abuse narrative used in the current study has also been used in previous studies using the simulated memory paradigm (Hong & Hobbs, 2021; McWilliams et al., 2014; Newton & Hobbs, 2015). The narrative is written from the second person perspective to allow participants to roleplay being the main character (e.g., “You are trying to fall asleep when Tom enters the room”). In the narrative, the main character spends a day with two friends, Chris and Jamie, and Jamie's older brother Tom (abuse‐unrelated information). The main character spends the night at Jamie's house, where eventually the (sexual) abuse happens at night when the older brother enters the boys' bedroom (abuse‐related information).

#### 2.2.2 Session 1: Emotional involvement questionnaire

The emotional involvement questionnaire (EIQ) was developed by Christianson and Bylin (1999) to measure emotional involvement in the perpetrator role play scenario they used for simulated amnesia work. Upon adapting this paradigm to a CSA narrative and victim perspective, McWilliams et al. (2014) used the EIQ in the SMEP paradigm. This has also been adapted by studies employing the SMEP since (Hong & Hobbs, 2021; Newton & Hobbs, 2015). The EIQ has two scales; one is a self‐report measurement of how emotionally involved the participant feels with the narrative (rated from *0 not at all* to *10 very much*) and the other is a measurement of how emotionally influenced the participant feels (*0 extremely negative* to *10 extremely positive*).

#### 2.2.3 Session 1 and 2: PANAS

The Positive And Negative Affect Schedule (PANAS) is a brief measure of positive and negative affect that consists of two 10‐item mood scales that are rated on a five‐point Likert scale from very slightly or not at all (1) to extremely (5; Watson et al., 1988). The PANAS can be used as measures of affect for different time periods and was used in the current study to measure affective trait (i.e., instruction: generally). The PANAS scales show good internal consistency (Cronbach's *α* = 0.88 for the PA scale and *α* = 0.87 for the NA scale as a trait measure). The PANAS‐trait was used for exploratory purposes.

#### 2.2.4 Session 2: Source memory test

During the Source Memory test, participants were asked (1) whether they remembered discussing specific details in the police interview and (2) if they remembered that these details happened on the day of the narrative. Information from the source memory test was split into abuse related and unrelated information. In total, there were 29 details in the source memory test (i.e., four correct details discussed in the interview—two abuse related and two abuse unrelated; four correct details not discussed in the interview—two abuse related and two abuse unrelated; four details from the interview that were unrelated to the narrative—two abuse related, two abuse unrelated; four completely new, unrelated details—two abuse related, two abuse unrelated; four misinformation details from the interview (two abuse related and two abuse unrelated), and eight misinformation details from the eyewitness narrative (four abuse related and four abuse unrelated). Information from the narrative was divided into “abuse‐related” and “abuse‐unrelated” information by whether information stemmed from paragraphs of the narrative that described the day‐time, before the abuse happened, or from the paragraph during which the abuse is described. This distinction between where information from the story is abuse‐related or not was also described by McWilliams et al. (2014) who used a similar narrative. Similarly, misinformation given about earlier paragraphs in the story was deemed “abuse‐unrelated” whereas misinformation given about later paragraphs, during which abuse was described, was deemed “abuse‐related.”

#### 2.2.5 Session 2: COPE

The COPE is a 60‐item inventory that assesses people's coping responses to everyday stressors on 15 subscales, including scales such as humor, denial, or active coping (Carver et al., 1989). Items include “I make fun of the situation” or “I pretend it is not really happening” and are scored on a four‐point Likert scale ranging from (1) *I don't usually do this at all* to (4) *I usually do this a lot*. The cope was administered for exploratory purposes.

### 2.3 Design and procedure

The current study employed a between‐subjects design with group (false denial vs. honest control) as the independent variable. All participants received misinformation about the narrative and interview. The dependent variables were the number of correct items (four abuse related and four abuse unrelated) and misinformation items (three abuse unrelated and one abuse related) endorsed from the interview in the source memory test (i.e., interview true and false memory scores), and the number of correct items (two abuse related and two abuse unrelated) and misinformation items (four abuse unrelated, four abuse related) endorsed from the narrative in the source memory test (i.e., narrative true and false memory scores). Since the recall task and simulated police interview during session one constituted our experimental manipulation, we did not conduct analyses on these. *Yes* responses on the memory tests were scored as (1), and *No* responses were scored as (0). Summary scores were calculated for the different dependent variables and transformed into proportions (range: 0–1).

**FIGURE 1:**
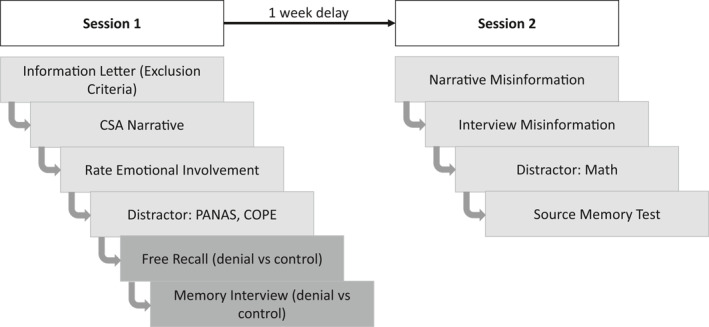
Diagram of the study procedure

The experiment took place online via Qualtrics, in two sessions that were divided by a 1‐week delay (Figure 1).

#### 2.3.1 Session 1

Before starting the first session, participants received an information letter about the study and were asked to provide written informed consent. As part of the information letter, we included a cautionary note asking participants who have experienced certain trauma not to participate, to prevent potentially negative effects for participants (McWilliams et al., 2014). Next, participants received the child sexual abuse narrative and were asked to read it twice, imagining being the main character and empathizing with the role. Then, participants rated their emotional involvement on the EIQ. Subsequently, participants filled in the PANAS trait and COPE questionnaire, as a distractor task. Then, the manipulation phase occurred: participants who had been randomly assigned to the false denial condition were instructed to recall the day described in the narrative to a friend by being truthful about what happened during the day, but explicitly denying that any abuse happened at night. Specifically, participants received the following instructions: “*You are not ready to discuss that you are a victim, so you do not want to tell all of the details. Recall information from the day truthfully, but* ***explicitly deny that any abuse happened***” (see https://osf.io/9c5j4/?view_only=dcb3d7c42fb24e33afc030f326294061 for complete instructions). Alternatively, participants in the honest control condition were instructed to recall the entire day and night truthfully to a friend, disclosing the abuse. After this free recall, participants engaged in a memory interview which contained 24 yes/no style questions, 16 of which were related to the narrative (true details; 10 abuse unrelated and 6 abuse related) and 8 of which were unrelated (4 abuse related and 4 neutral). Participants were told that the police was notified, and that the memory interview they completed was a simulation of a police interview. Those in the false denial group were asked to answer questions about the day truthfully, but to continue denying that any abuse happened, whereas control participants were instructed to answer questions as honestly as possible. The memory interview terminated the first session.

#### 2.3.2 Session 2

After a 1‐week delay, participants received an email with a link inviting them to complete the second session via Qualtrics. First, all participants were told that as part of the routine of being interviewed by the police, they would receive a summary of the police interview that was conducted with them in the first session. This summary actually included four pieces of misinformation regarding which details were discussed. Then, participants were told that Jamie had been contacted as a potential witness, and that he came forward saying that he actually witnessed the abuse incident but had not said anything because he was scared. Participants received Jamie's witness account of the entire day and night. This narrative also included misinformation—four misinformation details regarding the abuse information, and four regarding the non‐abuse information. Subsequently, participants engaged in a math distractor task. Finally, they completed a source memory test checking their memory for both the interview and narrative during which all participants were instructed to respond truthfully. At the end of the second session, participants were thanked and debriefed.

## 3 RESULTS

We used a statistical significance cut off score of alpha = 5% for all analyses. Analyses were conducted on all details, and then split up for abuse related and abuse unrelated details. We additionally conducted non‐parametric Mann‐Whitney U tests for variables which were non‐normally distributed to check that there would be no false‐positive results, even though our sample was large enough to deal with slight skewness. Effect sizes for the Mann–Whitney *U* test are given as Rank Biserial Correlations (*r*). There were no extreme outliers (>3 SD away) for any of the variables.

### 3.1 Memory for the narrative

#### 3.1.1 True memory

One sided independent samples Welch's *t*‐tests were performed to compare the proportion of participants' correctly endorsed information from the narrative. Overall, participants in the honest control group and false denial group did not differ statistically significantly in their endorsement of true information, *t*(100) = 0.9, *p* = 0.2, *d* = 0.16 (Table 1).

**TABLE 1:** Participants' means and standard deviations for true memory for the narrative

Condition	Total		Abuse related		Abuse unrelated	
	*M*	*SD*	*M*	*SD*	*M*	*SD*
False denial	0.77	0.17	0.85	0.30	0.74	0.21
Honest control	0.80	0.16	0.91	0.16	0.75	0.16

Looking at reports for the abuse‐related paragraph of the narrative separately we found that participants in the two experimental groups did not statistically significantly differ with respect to their endorsement of abuse‐related information, *t*(67) = 1.5, *p* = 0.07, *d* = 0.28. Considering true memory for the abuse unrelated information from the narrative, participants in both groups endorsed almost the same amount of true information and there was no statistically significant difference, *t*(104) = 0.23, *p* = 0.41, *d* = 0.04 (see Table 1).

#### 3.1.2 False memory

**FIGURE 2:**
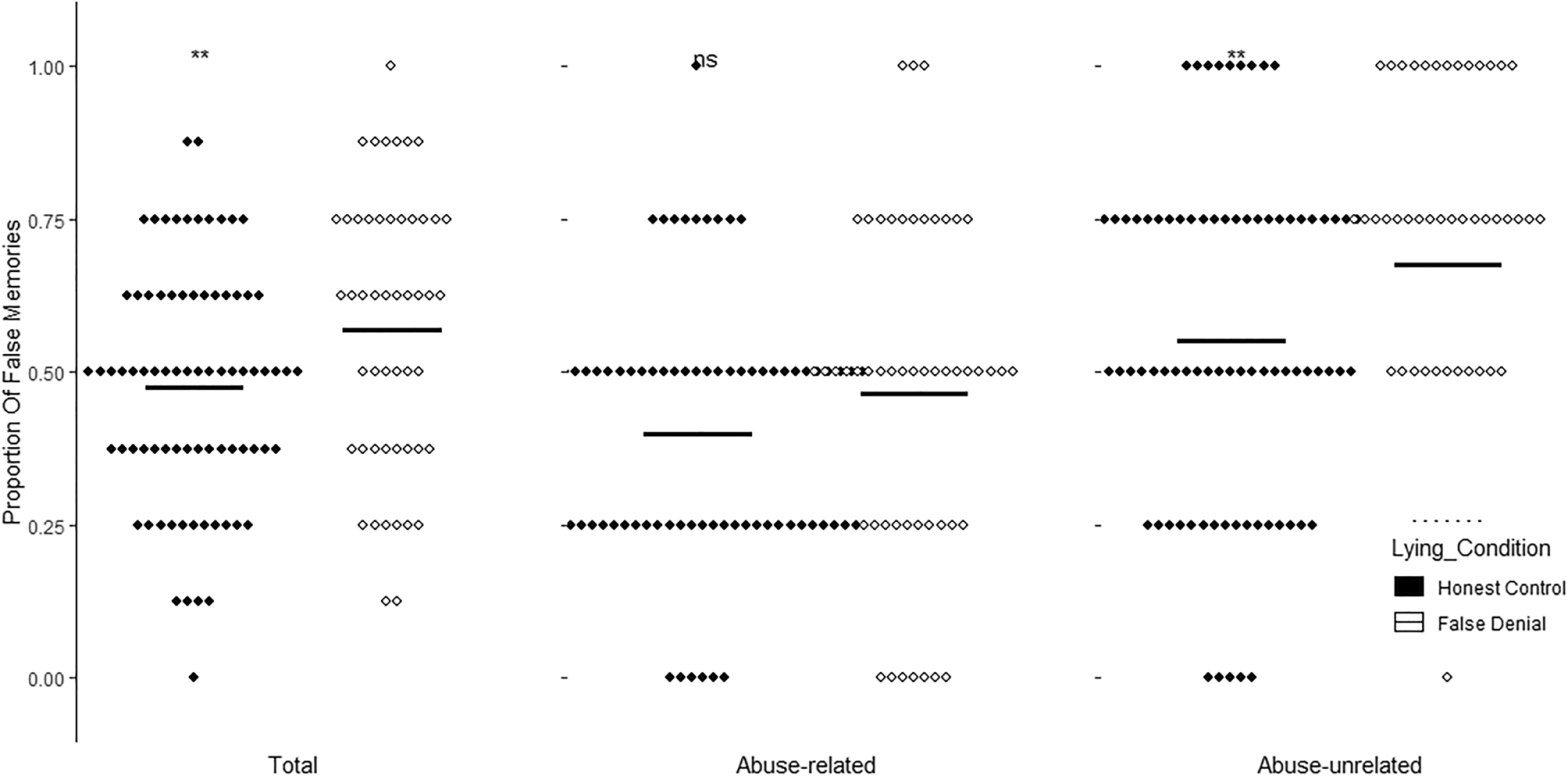
Participants' misinformation endorsement for the narrative. Each dot represents one participant. Means are represented by bars. ** = *p* < 0.05, ns = *p* > 0.05

We conducted two‐sided independent samples student *t*‐tests to compare the amount of false information that participants in the honest control and false denial conditions endorsed from the misinformation they received about the narrative. Participants in the false denial condition endorsed statistically significantly more false information (*M* = 0.57, *SD* = 0.23) from the co‐witness misinformation as compared with participants in the honest control condition (*M* = 0.48, *SD* = 0.19; see Figure 2), *t*(125) = −2.53, *p* = 0.013, *d* = 0.46. The difference between the two groups is around a proportion of 0.1 which is roughly equivalent to one false information detail. Because the data were skewed (honest control: *W* = 0.96, *p* = 0.014; false denial: *W* = 0.95, *p* = 0.021), we additionally performed a non‐parametric Mann–Whitney *U* test, replicating these findings, *W* = 1449, *p* = 0.017.

Investigating the abuse related misinformation separately, we found that participants in the false denial (*M* = 0.47, *SD* = 0.28) and honest control groups (*M* = 0.40, *SD* = 0.21) did not differ statistically significantly in misinformation endorsement of false abuse‐related information, *t*(125) = −1.51, *p* = 0.133, *d* = 0.27 (Figure 2).

Yet, when considering abuse‐unrelated misinformation, participants in the false denial group (*M* = 0.68, *SD* = 0.27) endorsed statistically significantly more false information than participants in the honest control group (*M* = 0.55, *SD* = 0.28), *t*(125) = −2.48, *p* = 0.014, *d* = 0.45 (Figure 2) from the narrative misinformation. Because this data were also skewed (honest control: *W* = 0.91, *p* < 0.001; false denial: *W* = 0.88, *p* < 0.001), we additionally performed a non‐parametric Mann‐Whitney *U* test, which again replicated the findings, *W* = 1448.5, *p* = 0.015.

### 3.2 Exploratory analyses

We additionally performed some (not preregistered) exploratory correlation analyses to investigate whether true and false memory (misinformation endorsement) scores were related to each other for both the false denial and honest control groups. For all information from the narrative, we found no statistically significant correlation between true and false memory scores for the narrative for both honest control: *n* = 77, *r* = −0.18, *p* = 0.12, and false denial participants: *n* = 50, *r* = 0.04, *p* = 0.81. When looking at only non‐abuse related details from the narrative, we found no statistically significant correlations either (i.e., honest control group: *n* = 77, *r* = −0.2, *p* = 0.09; false denial group: *n* = 50, *r* = −2.1, *p* = 0.15).

**FIGURE 3:**
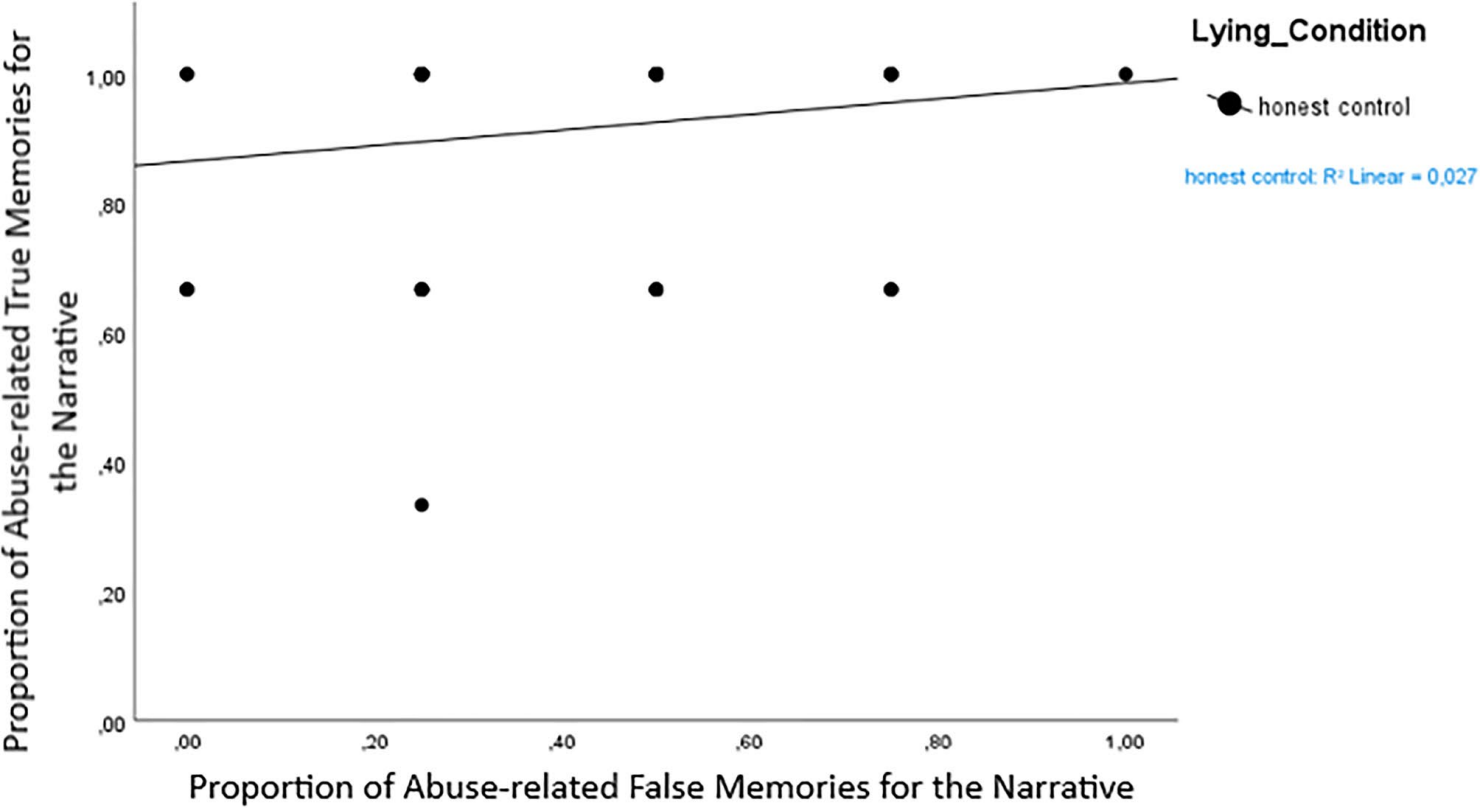
True and false memories for abuse‐related details of the narrative in the honest control group. Scores are given in proportions

**FIGURE 4:**
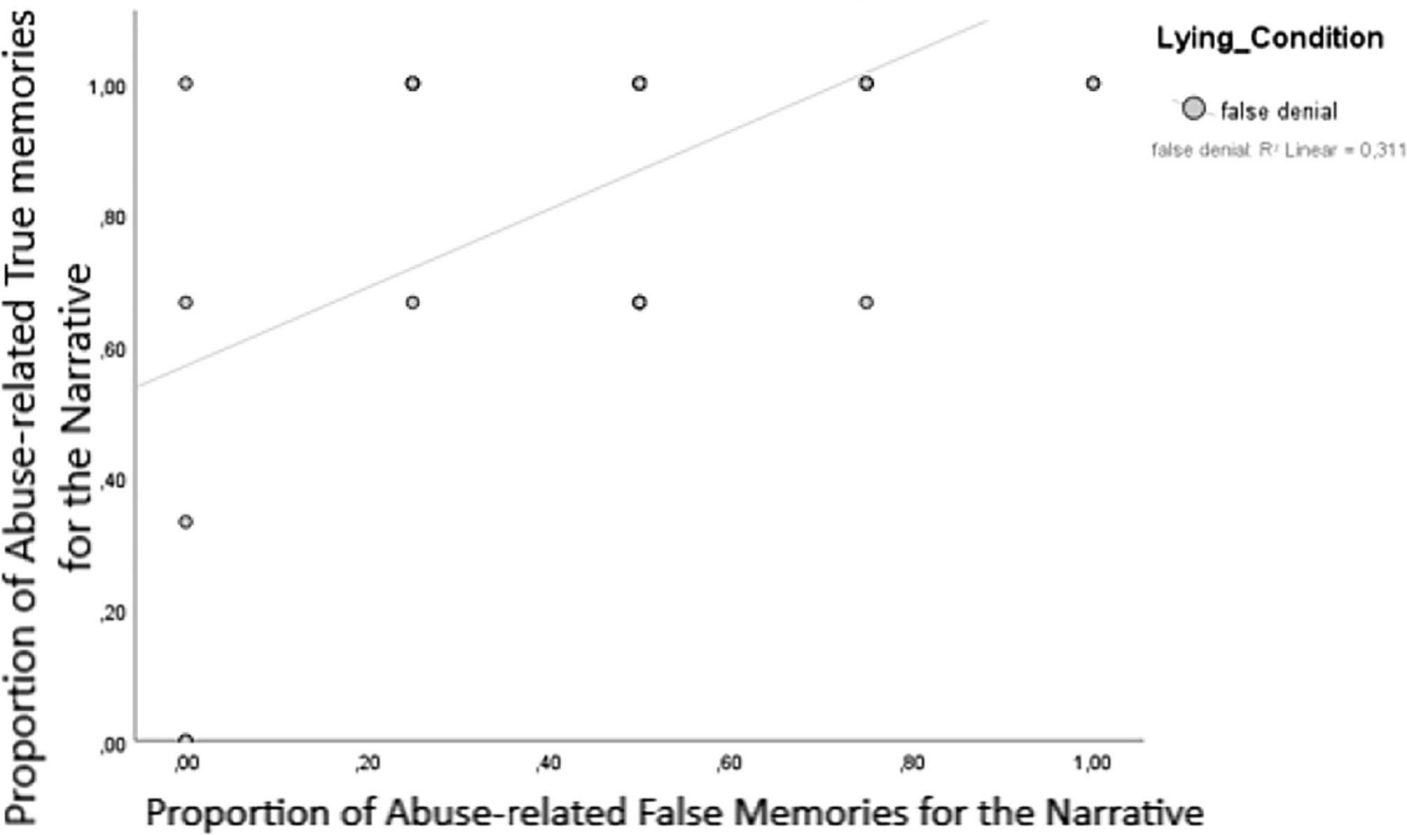
True and false memories for abuse‐related details of the narrative in the false denial group. Scores are given in proportions

However, when we examined correlations between true and false memory scores for the abuse related information independently (which was also the information that was denied), we found a statistically significant correlation between true and false memory scores in the false denial condition (*n* = 50, *r* = 0.56, *p* < 0.001, Figure 3), but not for participants in the honest control condition (*n* = 77, *r* = 0.16, *p* = 0.16, Figure 4).

### 3.3 Memory for the interview

#### 3.3.1 True memory

Two‐sided independent samples student's *t*‐tests were performed on the true memory data for the interview. For all interview details, participants in the false denial and honest conditions scored very similar, and no statistically significant differences could be found, *t*(125) = −0.38, *p* = 0.71, *d* = 0.07 (Table 2). When looking specifically at endorsement of the abuse‐related information (information which was denied during the interview phase), scores of participants in the two experimental groups did not statistically significantly differ from each other, *t*(125) = 1.02, *p* = 0.31, *d* = 0.19 (Table 2).

**TABLE 2:** Participants' means and standard deviations for true memory for the interview

Condition	Total		Abuse related		Abuse unrelated	
	*M*	*SD*	*M*	*SD*	*M*	*SD*
False denial	0.78	0.18	0.86	0.18	0.69	0.28
Honest control	0.76	0.21	0.89	0.17	0.63	0.32

Regarding endorsement of true information that was unrelated to the abuse information and not denied during the interview phase, there was no statistically significant difference between the two groups, *t*(125) = −1.09, *p* = 0.28, *d* = −0.2 (Table 2).

#### 3.3.2 False memory

A two‐sided independent samples student's *t*‐tests was conducted to compare the amount of false information (in proportions) from the interview misinformation that participants in the two conditions endorsed in the source memory test. We found no statistically significant difference in false memory endorsement in participants in the false denial (*M* = 0.33, *SD* = 0.2) and honest control (*M* = 0.31, *SD* = 0.23) groups for the interview misinformation, *t*(125) = −0.5, *p* = 0.62, *d* = −0.1.

## 4 DISCUSSION

The core aim of the present experiment was twofold. First, we wanted to assess whether the typical mnemonic effects of denial would be replicated in a simulated memory error paradigm, in which participants imagined being the victim of abuse. Second, we were interested in whether false denials (from such a victim perspective) would affect suggestion‐based false memories.

A new contribution of this work is that participants who falsely denied details of the abuse portion of the narrative were more likely to endorse false (non‐abuse) details from an eyewitness account than truth‐tellers. This difference was equivalent to around one information detail falsely endorsed more in the false denial compared to the honest control group. In other words, about 67% of the false denial data was above the mean from the honest participants' memory performance. This finding that false denials were related to higher suggestion‐based false memory endorsement is seemingly in contrast with previous research showing that false denials are related to lower spontaneous false memory rates in the DRM paradigm (Otgaar et al., 2020). However, research has shown that suggestion‐based false memories and spontaneous false memories are not necessarily related to one another, and studying false memories in different paradigms can lead to different results (Otgaar & Candel, 2011; Otgaar et al., 2017; Ost et al., 2013). Moreover, the current paper employed a victim perspective, whereas Otgaar et al. (2020) studied false denials and false memories from a witnessing perspective, which might impact memory effects differently (Bücken et al., 2022).

Our hypotheses surrounding misinformation endorsement were based on the premise that false denials would weaken true memory for the narrative as well as arguments from Fuzzy Trace Theory and Associative Activation theory (Howe et al., 2009; Reyna & Brainerd, 1998; Tousignant et al., 1986). Indeed, we expected that lower *true* memory scores for details after false denial would be associated with higher or lower (depending on the theory) *false* memory scores.

However, not in line with our expectations, we found no forgetting effects of false denials regarding the narrative in the current study. We expected to find such a forgetting effect because we employed complex false denials (i.e., only the critical part of the event was denied) of an event that was negative in valence (i.e., child sexual abuse). Previous studies that employed either complex denials or false denials of a negative emotional experience have found a (small) forgetting effect of information from the initial stimulus after false denial (e.g., Battista et al., 2021; Otgaar et al., 2020; Romeo et al., 2019). Memory for the denied event is not always found to be affected by denial, and there are several studies that did not find such an effect either (e.g., Otgaar et al., 2014, 2016, 2018). Arguably, a reason that we did not find this effect is because we employed the victim perspective.

Because we did not find an effect of false denial on forgetting of details related to the narrative, it is likely that our false memory results were not driven by true memory performance. In line with this, we found no statistically significant correlation between true and false memory scores (when looking at abuse related and unrelated details together), as we expected in our predictions of false memory endorsement with a single exception (i.e., for abuse‐related details which we will discuss below).

Therefore, neither Fuzzy Trace Theory nor Associative‐activation theory can fully account for how false denials might be related to the increased false memory scores we found in the current experiment. According to Fuzzy Trace Theory, false memories arise when our memory for details (i.e., the verbatim memory trace) is weak and we rely on our gist memory to recall an event (Reyna & Brainerd, 1998). False denials would fit into this theory if the denial was associated with weaker memory for details. However, because we did not find that false denials influenced true memory for the narrative, Fuzzy Trace theory fails to explain why we found an increased misinformation effect. Similarly, according to Associative‐activation Theory, when true memory for an event is weaker or inhibited, information stored in relevant related nodes (e.g., related misinformation) might not be retrieved either (Howe et al., 2009) so that false memory might also be lower. However, we did not find lower true nor lower false memory scores for falsely denying participants compared to consistently honest ones in the current experiment.

There was one exception to this: We found that for participants who falsely denied the abuse, true and false memories *for the abuse* were correlated quite strongly (*r* = 0.56, *p* < 0.001) in that false deniers who had lower true memory scores also had lower false memory scores for the denied abuse information. This was not the case for honest participants or for the non‐abuse related information. The positive correlation shows that the extent to which the denial impacted true memory (i.e., and lead to retrieval inhibition; Otgaar & Baker, 2018; Otgaar et al., 2020) was linearly related to the extent to which related pieces of misinformation were impacted by the denial. This is in line with the tenets of Associative‐activation theory; assuming that misinformation is associatively related to details of the true memory experience (as it was in our experiment), activation of true memory details might spread to the activation of misinformation details in a memory network (Howe et al., 2009). Similarly, building on this theory, we might assume that inhibition of memory nodes related to the experience might limit the spread of activation to associated memory nodes of the misinformation, thereby lowering false memory production (see Otgaar et al., 2020). Thus, Associative‐activation theory would lead us to assume that there should be a positive correlation between true and false memory scores, which is in line with the correlation we found in falsely denying participants for abuse‐related information from the narrative. So, it does seem that denials impacted the memory network, perhaps by inhibition processes affecting the lied‐about information, even though no clear forgetting effect after denial emerged.

It could also be the case that after denial of the abuse, participants were less confident in their memory of the abuse (i.e., false denial affected their beliefs about the abuse), as it has also been suggested by Otgaar and Baker (2018). It is possible that participants then adopted a specific response criterion in line with their individual general tendencies that they employed to *report* their memories (i.e., in line with Signal Detection Theory; Wixted, 2007). Participants who were more liberal answered “yes” more often to recognition questions about the abuse (true memory and misinformation questions), indicating that they were old, whereas more conservative participants answered “no” more often to recognition questions about the abuse, indicating that they were new (Wixted, 2007). Thus, it is possible that—after denial—participants had a response bias *for information relevant to the denial (*i.e.*, abuse‐related information)* and relied more heavily on a specific response criterion than participants in the honest control condition. Collecting confidence ratings could have clarified this issue. However, because we did not collect data on confidence, we can only speculate at this point.

Surprisingly, when looking at false memories for the abuse portion and non‐abuse portion of the narrative separately, we found that denying the abuse did not lead to more endorsement of false memories of abuse related information, but of *abuse unrelated* information. This difference was not only statistically significant, but participants who falsely denied endorsed one more abuse‐unrelated piece of false information than honest participants. Thus, their statements might seem more inconsistent which is a sign sometimes used by the police to distrust an eyewitness statement (Fisher et al., 2013). Of course, because the abuse unrelated details never had to be denied, it is not surprising that “usual” memory distortion might occur. What is surprising is that participants who falsely denied abuse information endorsed more misinformation about non‐denied (i.e., abuse unrelated) information than honest participants.

A possible explanation for this goes back to the Discrepancy Detection Principle (Tousignant et al., 1986). Perhaps participants who falsely denied the abuse were more focused on the abuse related part of the narrative, trying to make sure that they would deny details related to the abuse while being honest about abuse unrelated information. It is possible that their attention was more focused on what they had to lie about (i.e., the abuse), rendering it less likely that they would detect discrepancies about the *abuse unrelated* details. According to the Discrepancy Detection Principle then (Tousignant et al., 1986), they would be more likely to endorse abuse‐unrelated misinformation.

Of importance, this finding that false denials are related to false memories of abuse unrelated but not abuse related information has implications for the legal field. Even though victims who deny their traumatic experiences might make more mistakes about events leading up to the abuse and misreport such details in their memory reports, their reports for the critical experience itself does not seem to be affected, at least in our study. Therefore, their testimony about the abuse itself—which arguably is the most critical aspect in court—might not be negatively affected by false denials.

Furthermore, we did not replicate the denial‐induced forgetting effect (for the interview). This is not in line with previous research on the mnemonic effects of false denials from a *witnessing* perspective (for a review, Otgaar & Baker, 2018; but also Battista et al., 2021; Otgaar et al., 2018; Romeo et al., 2019). However, a recent study has shown that the mnemonic effects of false denials might vary based on the perspective with which this type of lie is studied (Bücken et al., 2022). Indeed, in the current study, participants had to roleplay being the victim. This means that they were more involved in the story than an external witness and the denied information was more self‐referenced. This might be especially true for the interview in which questions were asked from the second person perspective (e.g., “Did your parents go with you to dinner that evening?”) and answered by participants from the first person perspective (e.g., “No, my parents were not at dinner that evening.”). Such self‐referenced information is better remembered than other‐referenced information, an effect known as the self‐reference effect (Symons & Johnson, 1997; Wang et al., 2019, 2021). This might explain why we failed to replicate DIF from a victim perspective. More research from the victim perspective is needed to investigate whether indeed, false denials from a victim perspective have different mnemonic effects.

Interestingly, we did not find an effect in which false denials impacted false memories for the interview. This finding is not in line with our hypotheses (which were the same as those for misinformation endorsement for the narrative), but not surprising in light of our findings regarding true memory for the interview. Because we did not find that memory for the interview was affected by false denial, it is not surprising that we did not find an increased interview misinformation effect for the denial group.

### 4.1 Limitations

Some limitations of the current study are worth mentioning. First, this experiment was fully conducted online. Thus, perhaps there was less control over how participants followed instructions. Furthermore, the “police interview” was conducted in a written form as opposed to a usual face‐to‐face setting. Indeed, one might argue that a written interview is not as ecologically valid as an in‐person interview, and that certain social dynamics (e.g., the authoritative position of the interviewer, non‐verbal social cues) are missing in such a setting. This might contribute to the fact that we were unable to replicate the effects surrounding memory for the interview seen in false denial studies (i.e., DIF). Future research should try to disentangle whether denial‐induced forgetting can be replicated in written interviews. Another limitation is related to our sample which consisted of undergraduate students who have not experienced trauma related to child abuse (as per the warning against participation in case they have experienced certain traumatic events in the past). For ethical reasons, we did not use a maltreated sample in our experiment. Studies show that maltreated and non‐maltreated individuals have quite similar cognitive processes (Goodman et al., 2011; Howe et al., 2004, 2011). However, our sample might still differ somewhat from the population of interest, because our participants “only” simulated being the victim of abuse.

## 5 CONCLUSION

We found that false denials of a simulated child sexual abuse narrative did not lead to forgetting of details of both the narrative and the simulated police interview during which the abuse was denied. Moreover, false denials of abuse led to increased false memories of abuse unrelated information that occurred before the abuse, but not increased misreporting of information directly related to the abuse. Thus, when a false denial is exercised from the perspective of a victim, the victim's memory reports of the critical abuse experience itself might not be tainted by their initial lie when they eventually do come forward.

## CONFLICT OF INTEREST

The authors declare that they have no conflict of interest.
